# Extended Trochanteric Osteotomy as a Tool in the Revision of Hip Arthroplasty Due to Aseptic Loosening

**DOI:** 10.7759/cureus.71557

**Published:** 2024-10-15

**Authors:** René Gutiérrez-Jiménez, Felipe Delgado-Cantú, Carlos E González Rivera, Alan Monroy-Quiroz, Fernanda P Pons-Faudoa

**Affiliations:** 1 Traumatology and Orthopedics, Hospital San José de TecSalud, Monterrey, MEX; 2 Traumatology and Orthopedics, Hospital Christus Muguerza Alta Especialidad, Monterrey, MEX; 3 Traumatology and Orthopedics, ISSSTE (Instituto de Seguridad y Servicios Sociales de los Trabajadores del Estado) Clinica Hospital Constitución, Monterrey, MEX; 4 Medicine, Tecnologico de Monterrey, School of Medicine and Health Sciences, Monterrey, MEX

**Keywords:** aseptic loosening, cement, component exchange, extended trochanteric osteotomy, revision surgery, total hip arthroplasty, total hip replacement

## Abstract

Aseptic loosening of femoral and acetabular components is a common complication following total hip arthroplasty (THA). It presents a significant diagnostic and therapeutic challenge for orthopedic surgeons, as it requires differentiation from infection and often necessitates complex revision surgery.

We present the case of a 76-year-old female with a surgical history of total right hip arthroplasty performed one year prior. The patient presented with pain and limited mobility in the right lower extremity. Laboratory studies, including inflammatory markers and cultures, were negative for local or systemic infection. A biopsy of the right hip confirmed the absence of infection. Given these findings, a diagnosis of aseptic loosening was made. A revision right hip arthroplasty was undertaken, during which the acetabular component was found to be intact and was therefore retained. New femoral components were placed using an extended trochanteric osteotomy approach. Postoperatively, the patient demonstrated significant clinical improvement, regaining the ability to walk without support and exhibiting improved range of motion in the affected limb.

Aseptic loosening of THA often requires revision surgery with component exchange to avoid any further complications that can increase morbidity and mortality. This produces a diagnostic and therapeutic challenge for the orthopedic surgeon due to the difficulty of cement retraction, previous implants, and the placement of new prosthetic components. Extended trochanteric osteotomy is an excellent tool for the orthopedic surgeon facing a revision of THA to remove the cement mantle and femoral stem and avoid complications.

## Introduction

Total hip arthroplasty (THA), also known as total hip replacement, is a highly effective surgical procedure commonly performed to alleviate pain and restore function in patients with hip joint diseases, such as osteoarthritis, several types of fractures, rheumatoid arthritis, and avascular necrosis. Since its inception in 1960, THA has significantly improved the quality of life for millions of patients worldwide, boasting success rates of more than 90% at 10-15 years postoperatively [1].

Despite the overall success of THA, complications can occur, with aseptic loosening being one of the most prevalent and challenging. Aseptic loosening refers to the failure of the bond between the bone and the implant in the absence of infection. It typically results from the wear and tear of the implant materials, leading to osteolysis, bone resorption, and, ultimately, implant loosening [2]. Osteolysis is the most common cause of aseptic loosening of THA [3]. Patients who are young, obese or have high activity levels are at an increased risk for clinically significant aseptic THA loosening [4-6]. This complication remains a significant cause of pain and disability, often necessitating revision surgery [7].

Diagnosing aseptic loosening is complex and requires a thorough evaluation to exclude infection, which can present with similar symptoms. Laboratory tests, imaging studies, and sometimes biopsies are essential to differentiate between aseptic loosening and periprosthetic joint infection (PJI) [8]. Treatment often involves revision surgery, which can be technically demanding due to the need for removal of well-fixed components, management of bone loss, and placement of new prosthetic components [9].

Understanding the risk factors, diagnostic methods, and treatment options for aseptic loosening is crucial for orthopedic surgeons to manage this complication effectively and improve patient outcomes. The objective of this case report is to highlight the diagnostic challenges of aseptic loosening in the context of a revision THA and to underscore the clinical utility of extended trochanteric osteotomy (ETO) as a valuable surgical technique for managing complex cases.

## Case presentation

A 76-year-old female patient with no known comorbidities presented with persistent pain in her right hip and thigh, accompanied by reduced mobility in the affected limb. She reported experiencing increased pain when bearing weight on the right leg. The patient had undergone a total right hip arthroplasty at a different medical facility one year prior due to severe coxarthrosis. Unfortunately, the surgical intervention report from the initial procedure was not available for review despite requests.

The patient consulted an orthopedic surgeon for persistent pain in her right knee, hip, and thigh without any recent history of trauma. During the physical examination, she exhibited pain in the right hip and thigh, reduced mobility in the affected limb, and increased pain with weight bearing, but no rotational deformity. Specific hip examination maneuvers revealed a positive Patrick test, a positive Stinchfield test, and a positive impingement test on the right hip. The range of motion was notably limited: flexion at 75°, extension at 10°, abduction at 25°, adduction at 20°, external rotation at 20°, and internal rotation at 5°.

**Figure 1:**
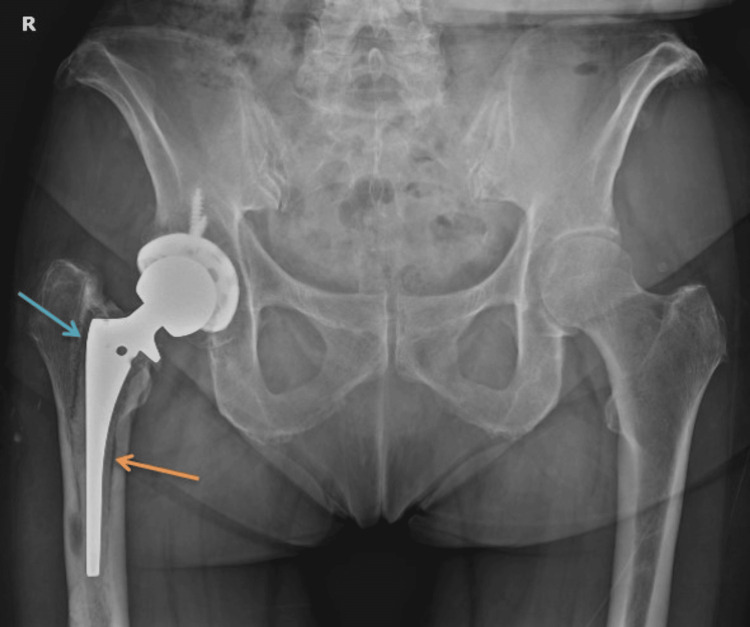
Anteroposterior X-ray of the pelvis. Arrows indicate implant-cement interface. Blue arrow demonstrates radiolucency between the stem and cement mantle in Gruen zone 1. Orange arrow demonstrates radiolucency between cement mantle and bone.

Examination of the knee showed negative results for both the McMurray and Lachman tests, but there was mild pain at the joint line with no signs of infection. An anteroposterior hip X-ray (Figure 1) demonstrated radiolucency at the implant-cement interface in zones 1 to 7 of Gruen, indicating loosening of the femoral component without migration. There were no signs of loosening of the acetabular component.

**Figure 2:**
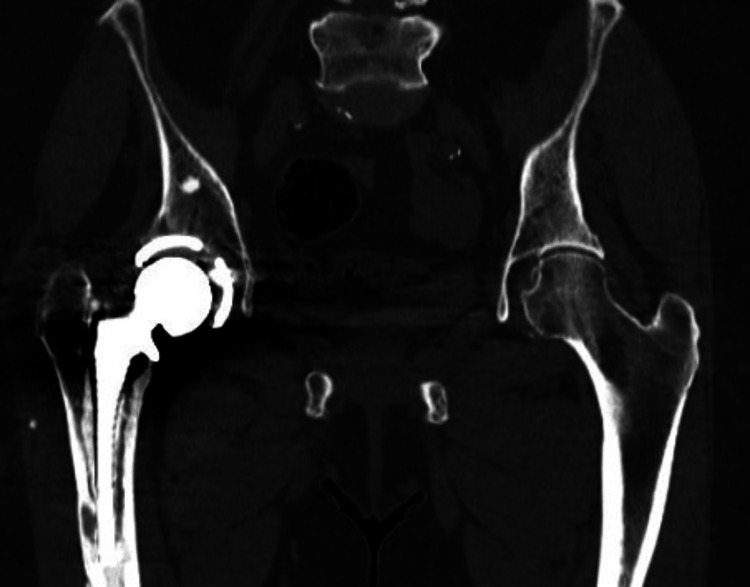
Pelvic non-contrast CT scan in coronal view. Hypodense zones between cement mantle and bone in zones 2, 3, 5, 6, and 7 of Gruen, and hypodensity in the superolateral third of the stem and the cement mantle.

A non-contrast pelvic CT scan (Figure 2) confirmed the loosening at the bone-cement interface, suggesting possible component loosening. Additionally, a hypointense oval area lateral to the distal end of the femoral stem implant raised suspicion of potential infection. However, preoperative blood analysis was within normal ranges, showing no marked leukocytosis, with a C-reactive protein (CRP) level of 0.6 mg/dL and an erythrocyte sedimentation rate (ESR) of 23 mm/hr.

Initially, a biopsy of the affected hip was performed under ultrasound guidance prior to definitive surgery. The biopsy revealed only scar tissue with no signs of infection, and the cultures were reported as negative.

In the second surgical stage, we proceeded with a revision surgery for THA. An anterolateral approach (Watson-Jones) was used, involving a 10- to 15-cm longitudinal incision. Careful dissection was carried out, paying particular attention to the gluteus medius, gluteus maximus, and tensor fasciae latae muscles. A T-shaped capsulotomy was performed, and the femur was dislocated to expose the acetabular component, which showed no signs of loosening or infection. However, the femoral component displayed clear signs of loosening and an unstable femoral stem.

**Figure 3:**
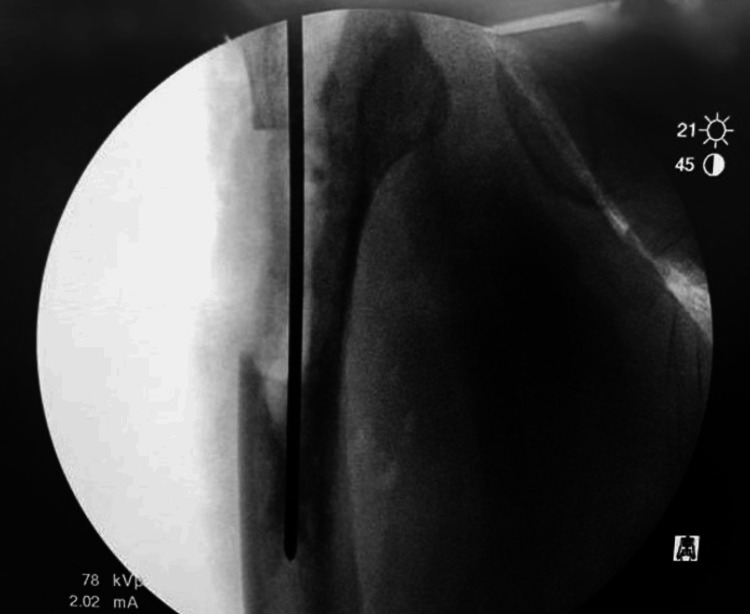
Fluoroscopy of the right proximal femur showing extended trochanteric osteotomy of the right femur.

A 10-cm extended proximal femoral osteotomy was performed on the anterolateral cortex of the right proximal femur, just below the greater trochanter, after hip dislocation (Figure 3).

This facilitated the removal of the femoral implant and cement. The attachments of the gluteus medius and vastus lateralis were preserved. Cuts were made on the anterolateral cortex of the proximal femur using an oscillating saw. Perforations were created in the marked osteotomy area with a high-speed burr, and an osteotome was used to complete the cortical opening. The cement mantle was removed with osteotomes, and the femoral stem was extracted manually. The femoral canal was then debrided and lavaged with a high-pressure pulsatile lavage system.

**Figure 4:**
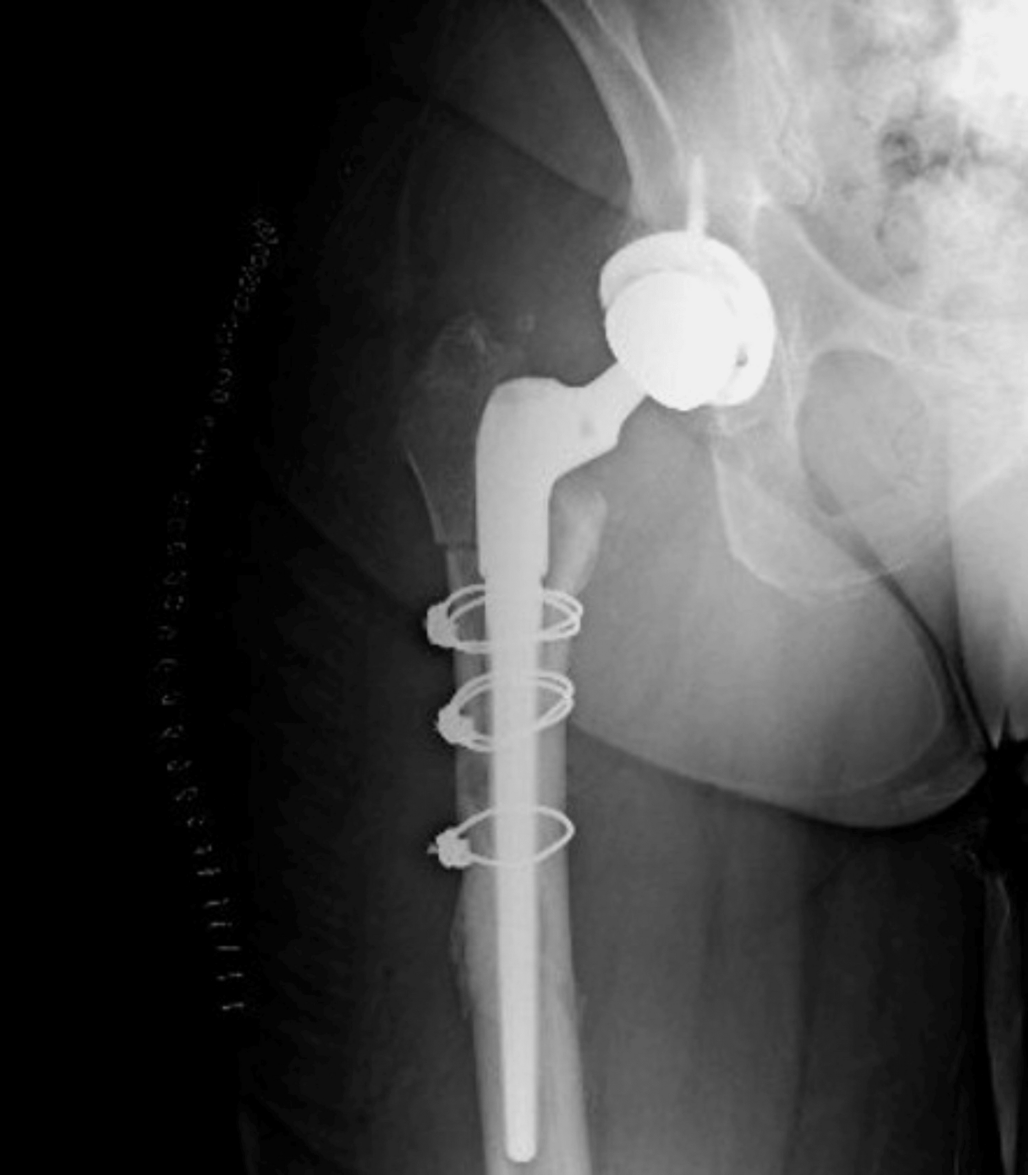
Postoperative anteroposterior X-ray of the right proximal femur demonstrating total hip revision arthroplasty result. A 36-mm cobalt-chrome femoral head, uncemented distal femoral stem 13 mm × 150 mm, and cone proximal body offset at 50 mm were used. To enhance stability, three cerclages of 1.8 mm × 914 mm were employed.

A new femoral head and an uncemented stem component were subsequently implanted. For this case, a 36-mm cobalt-chrome femoral head, a cone proximal body offset at 50 mm, and a 13 mm × 150 mm implant for the femoral stem were used. Upon completing the femoral component revision and prosthetic replacement, the proximal femoral osteotomy was fixed with three 1.8 mm × 914 mm cable cerclages (Figure 4).

After completing the procedure, soft tissue debridement and surgical wound irrigation are performed using a high-pressure pulsatile system. The capsule, muscle tissue, fascia, and subcutaneous tissue were closed with absorbable sutures, while the skin was closed with surgical staples.

Given the intraoperative assessment of the acetabular component, it was decided to maintain the existing implant intact. The procedure was carried out without complications, focusing on ensuring stability and functionality of the new femoral components.

After three months, the patient came for a check-up. The patient was walking without support or pain, with hip flexion of 110° and extension of 10°. The patient also had 35° of abduction and 10° of adduction. The surgical wound was well-healed.

**Figure 5:**
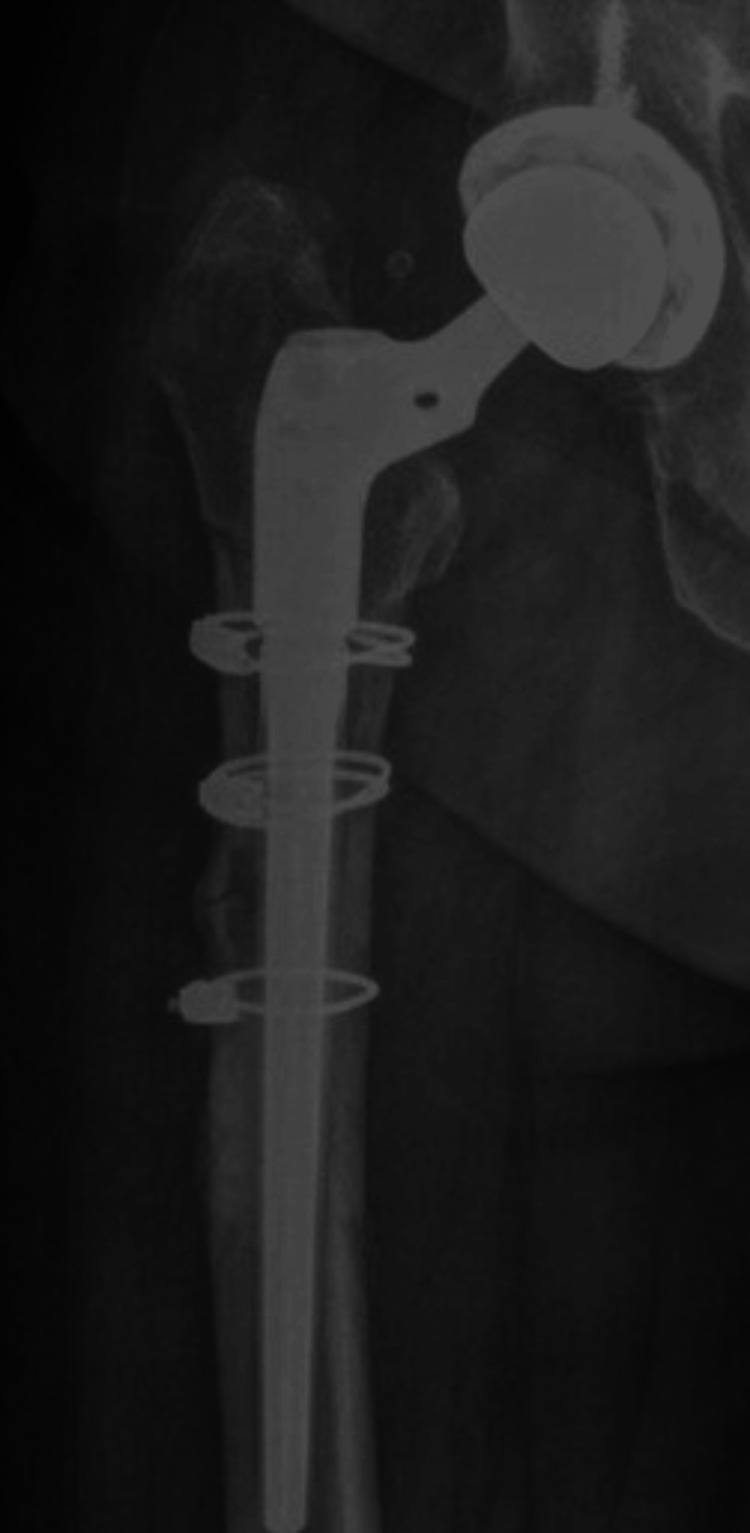
Three-month postoperative anteroposterior X-ray of the right hip. The image shows stable femoral component, osteotomy with evidence of bone healing, no evidence of loosening, and no signs of fracture or migration of the femoral stem and well-fixed implants.

A control anteroposterior radiograph of the pelvis was taken, showing a stable femoral component and an osteotomy with evidence of bone healing. There were no signs of fracture or migration of the femoral stem (Figure 5).

## Discussion

Aseptic loosening is the most common cause of revision surgery in THA, accounting for 35.1% of all cases [10]. Aseptic loosening particularly affects younger, obese or highly physically active patients [3-6]. Osteolysis, predominantly caused by polyethylene wear particles, remains the primary etiology, leading to advancements in materials such as ultra-high-molecular-weight highly cross-linked polyethylene to mitigate this issue [11,12]. Stress shielding, resulting in decreased cortical thickness, is also recognized as contributing to aseptic loosening [3,13].

Pain is a common but nonspecific symptom of aseptic loosening. Groin pain typically indicates acetabular component loosening, while thigh pain suggests femoral component involvement. A characteristic pain pattern involves initial discomfort with weight-bearing that diminishes as the implant stabilizes [13].

Radiography is the primary diagnostic tool for aseptic loosening in THA, revealing radiolucent lines at the interface between the prosthesis and bone in both anteroposterior and lateral views, classified by specific zones. The acetabular zones are categorized according to DeLee and Charnley, while the femoral zones are defined by Gruen. Serial radiographs are crucial for monitoring changes over time, including alterations in the position of the femoral stem relative to the calcar or shifts in the relationship between the femoral head and the greater trochanter. Additionally, the presence of bone formation distal to the implant, referred to as a pedestal, is indicative of loosening [11].

Laboratory investigations including ESR, CRP, and synovial fluid analysis are crucial to exclude infection. Given the progressive nature and risk of complications such as implant or bone fractures, surgical management is typically indicated. Treatment options for aseptic loosening in THA vary widely, ranging from debridement and bone grafting to more extensive interventions such as component replacement via revision arthroplasty [8-11]. The life expectancy and functional demands of the patient must always be taken into account.

After a careful clinical, radiographic, and laboratory examination confirms loosening of the femoral or acetabular implant, we can proceed with revision surgery if the patient is suitable. However, in cases in which the patient is not a candidate for revision surgery, such as in very elderly patients, those with low physical demand, or those with unfavorable general conditions that make major surgery risky, conservative treatment should be considered. Each patient's case must be individualized to determine the best course of action.

In cases in which the loosening affects the acetabular component, recommended approaches include component exchange, acetabular augments, cup-cage implants, antiprotrusio implants, and custom triflange implants, particularly in cases of severe bone loss. When dealing with loosening of the femoral component, revision femoral arthroplasty with noncemented fixation is generally preferred. The challenges of achieving stable interdigitation with polymethylmethacrylate cement in primarily cortical bone necessitate this approach [14].

ETO is a valuable technique in complex cases involving both septic and aseptic loosening, where removal of well-fixed cemented or uncemented stems is anticipated to be difficult. This procedure involves proximal femur osteotomy to facilitate cement mantle and stable stem extraction while reducing the risk of periprosthetic fractures, destruction of femoral bone stock, risk of bleeding, and surgical time, and correcting varus deformity of the proximal femur [15,16].

The most complicated and dangerous part of a hip arthroplasty revision is the removal of the cement mantle. Extreme care must be taken during this process to avoid femoral fracture, destruction of the bone stock, and cortical penetration. It is best to perform this step after revising the acetabular component, as removing the cement mantle can cause extensive bleeding from the medullary canal of the femur, which can obstruct the surgeon's view when inspecting the acetabular component [11]. In this case, we used ETO to assist the surgeon in removing the cement layer and femoral stem in cases of aseptic loosening in cemented total hip prostheses. This approach aims to achieve optimal outcomes during both the intraoperative and postoperative periods. Another option would be to remove the cement mantle via the medullary canal. However, studies have shown that there is an increased risk of extensive bleeding requiring blood transfusion, which has its own risk. Moreover, ETO has demonstrated better results with cement removal, and our experience in this case proved that it was the safest and best option [16].

## Conclusions

Surgical intervention is typically warranted in patients experiencing aseptic loosening following THA due to the condition's progressive nature, often leading to implant and/or bone fractures. The choice of surgical approach depends on the size and extent of the lesion, as well as the patient’s functional requirements and life expectancy, ranging from debridement and bone grafting with component retention to complete component exchange. Based on our clinical experience, our team advocates for the use of ETO to facilitate the extraction of cement and the femoral stem, thereby reducing the risk of periprosthetic fractures.
